# Curcumin, a Multifaceted Hormetic Agent, Mediates an Intricate Crosstalk between Mitochondrial Turnover, Autophagy, and Apoptosis

**DOI:** 10.1155/2020/3656419

**Published:** 2020-07-18

**Authors:** Nathan Earl Rainey, Aoula Moustapha, Patrice Xavier Petit

**Affiliations:** ^1^SPPIN, Saints-Pères Paris Neuroscience Institute, CNRS UMR 8003 Paris-City University, Campus Saint-Germain, Team “Mitochondria, Apoptosis, And Autophagy Signalling”, 45 Rue des Saints-Pères, 75006 Paris, France; ^2^Pharmacology and Toxicology Department, Faculty of Pharmacy, Homs., Syria

## Abstract

Curcumin has extensive therapeutic potential because of its antioxidant, anti-inflammatory, and antiproliferative properties. Multiple preclinical studies *in vitro* and *in vivo* have proven curcumin to be effective against various cancers. These potent effects are driven by curcumin's ability to induce G2/M cell cycle arrest, induce autophagy, activate apoptosis, disrupt molecular signaling, inhibit invasion and metastasis, and increase the efficacy of current chemotherapeutics. Here, we focus on the hormetic behavior of curcumin. Frequently, low doses of natural chemical products activate an adaptive stress response, whereas high doses activate acute responses like autophagy and cell death. This phenomenon is often referred to as hormesis. Curcumin causes cell death and primarily initiates an autophagic step (mitophagy). At higher doses, cells undergo mitochondrial destabilization due to calcium release from the endoplasmic reticulum, and die. Herein, we address the complex crosstalk that involves mitochondrial biogenesis, mitochondrial destabilization accompanied by mitophagy, and cell death.

## 1. The Structural Chemistry of Curcumin Related to Its Biological Effects

**Figure 1:**
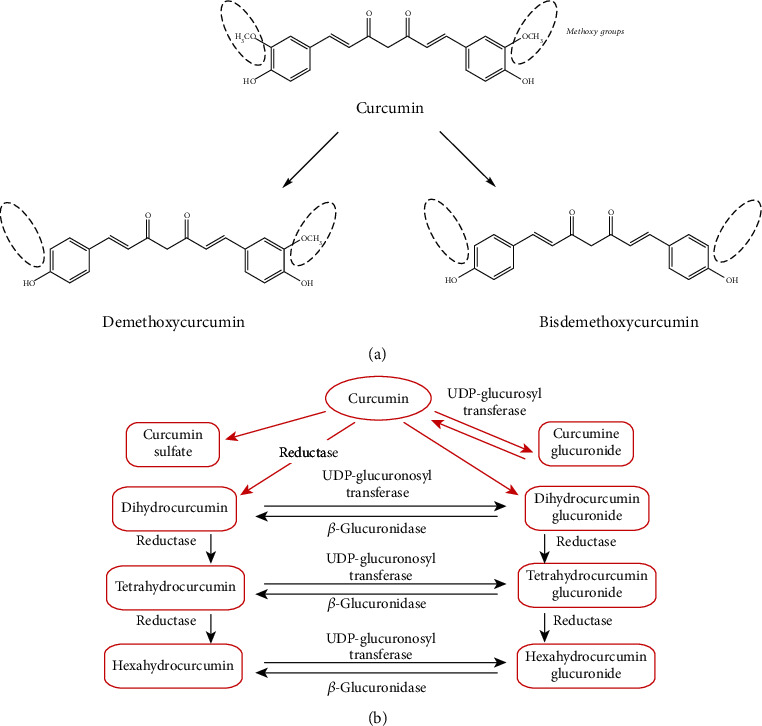
Curcumin structure and secondary metabolites. Schematic interpretation reproduced from Priyadarsini K.I. with permission [1] showing naturally occurring curcuminoids (a) and major metabolites produced once ingested (b).

Curcumin, or (1E,6E)-1,7-bis (4-hydroxy-3-methoxyphenyl)-1,6-heptadiene-3,5-dione, is a symmetric molecule also called diferuloyl methane (MW 368.38 g·mol^−1^) extracted from the dietary spice curcuma, historically used in Asian food and traditional medicine [1]. As previously described curcumin [2, 3] three chemical entities structure the molecule:2 o-methoxyphenol connected by a seven-carbon linker with an *α*,*β*-unsaturated diketone moiety (Figure 1(a)).The resonance structure inside the molecule is responsible for its participation in many electron transfer reactions.

**Figure 2:**
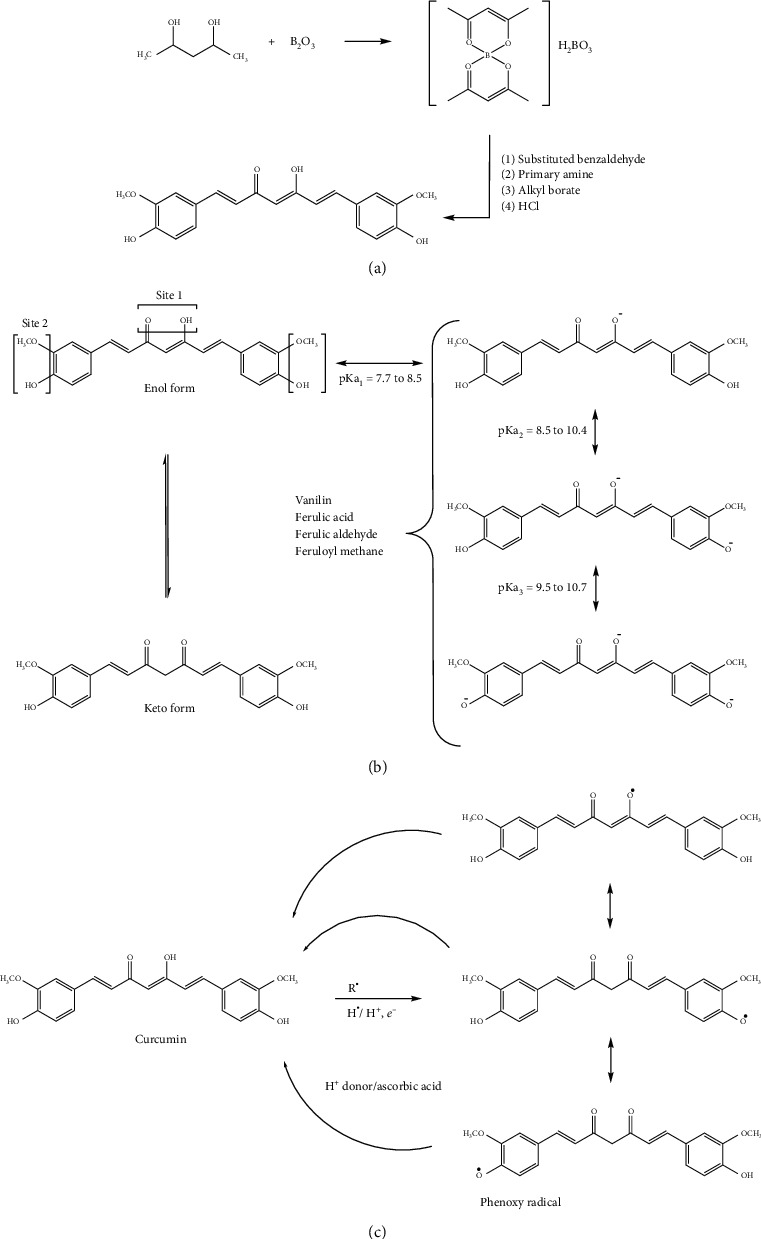
General consideration about the curcumin molecule. (a) Synthesis of curcumin by the general method proposed by Pabon [5]. (b) Keto-enol tautomerism (left) prototropic equilibria (right) and some of the degradation products of curcumin (center). (c) Possible sites of attack of reactive oxygen species with stabilization of phenoxyl intermediate and its regeneration by ascorbic acid or other proton donors. Schematic adapted with permission from Priyadarsini [1].

Almost a century after its isolation from turmeric, Lampe in 1913 [4] published the synthesis of curcumin in a series of steps starting with carbomethoxyferuloyl chloride and ethyl acetoacetate. Later, Pabon described a simple method for the synthesis of curcumin with high yields that is still in use today [5]. Patented processes indicating the use of B_2_O_3_, trialkylborate, and *n*-butylamine along with inert organic amide solvents have since improved yields [6–8] (Figure 2(a)). High-performance liquid chromatography (HPLC) is efficiently used for the detection and quantification of curcumin [9].

Curcumin is an electron donor and stabilizes its chemical structure by redistribution and resonance of the *π* electron cloud [1]. The extended conjugation confers UV-visible absorption properties (250-270 nm and 350-450 nm). So, curcumin fluoresces with emission starting at 470 nm. These optical properties are used for the isolation and purification of curcumin by various techniques and fluorescence which enable the monitoring of very low amounts of curcumin and related metabolites in plasma and urine in the range of 2.5 ng/mL [9–11]. Curcumin can also be excited at 488 nm with a lower fluorescent yield emission in the 500-550 nm range for detection in flow cytometry and confocal microscopy as we described before [2]. Curcumin is a weak Brönsted acid, with three labile protons and accordingly three pKa values—which can be estimated by both NMR and absorption spectrometry—corresponding to three prototropic equilibria (Figure 2(b)).

A key active site of curcumin for biological reactions is the diketo group, which acts as the primary hydrogen affinity site at physiological pH [1, 12]. The diketo group displays keto-enol tautomerism to reach prototropic equilibrium [12]. In addition to this enol (A-OH) site, the two phenol-OH locations appear to be more resistant to oxidation, but can undergo oxidation by electron transfer and hydrogen abstraction at more alkaline pH. Curcumin is a hydrophobic molecule with a log *P* value of 3.0 at neutral pH [13]. Therefore, curcumin is not easily soluble in physiological media and exhibits poor distribution and bioavailability [14].

As a whole, curcumin acts as a hydrophobic reducing agent and scavenges many reactive oxygen species (ROS) (Figure 2(c)) [14]. Phenoxyradicals formed can be regenerated by other H^+^ donors like ascorbic acid, for consecutive ROS elimination. Curcumin is as efficient in the removal of radicals as well-known antioxidants—thiols, vitamin A, vitamin C, and vitamin E—and mimics the function of superoxide dismutase [13].

The hydrogen donor site *α*,*β*-unsaturated *β*-diketo moiety is also considered the breakdown point in the curcumin structure, where curcumin hydrolysis and degradation take place essentially in aqueous media. Indeed, ninety percent of curcumin degrades within half an hour in water [15], giving rise by hydrolysis to several products, i.e., ferulic acid, ferulic aldehyde, and feruloyl methane.

The rate of curcumin hydrolysis significantly decreases when the diketo reaction site is complexed to lipids, peptides, proteins, surfactants, and other molecular structures, a situation occurring both in biological fluids and within cells [16]. Accordingly, curcumin solutions are more stable in culture media containing fetal calf serum (FCS), and evidence shows that once curcumin is in the bloodstream, hydrolytic degradation is abolished since the diketo function is occupied through binding to plasma proteins and other biomolecules [17]. On the other hand, even though hydrolytic degradation of curcumin scarcely happens *in vivo*, it is subject, once absorbed, to fast enzyme-mediated metabolism leading to hydrophilic metabolites [15]. Pathways have been proposed for the metabolism of hydrophobic curcumin to hydrophilic metabolites [13].

Curcumin is reduced to tetra-, hexa-, and octa-hydrocurcumins, and its two phenolic groups are conjugated to produce either curcumin glucuronide or curcumin sulfate [15] (Figure 1(b)).

The reduction or conjugation of curcumin is thought to be a slow process allowing curcumin to accumulate in cells and exert activity [18]. Glucuronidation of curcumin and curcuminoids, produced by the phase II detoxifying pathway in liver, results in the production of various curcuminoid-glucuronides with reduced activity [19]. However, as phase I enzymes are more expressed than phase II, curcumin absorption in liver induces transcriptional responses that enhance the antioxidant capacity of hepatic and extrahepatic tissues, which could explain some of curcumin's chemopreventive properties.

Other properties arise from a possible nucleophilic addition reaction between the unsaturated ketone of curcumin and anions like A-OH, A-SH, and A-SeH from other molecules [13]. As a result, curcumin can bind to various proteins. For instance, conjugation with glutathione-SH results in the depletion of the glutathione pool and the antioxidant defense system (ADS) in cells. In this regard, the depletion of glutathione molecules suggests that curcumin acts as a prooxidant contributor in some conditions [15]. But, depending on the concentration, this stress triggers an adaptive response boosting the glutathione production and other components of ADS. Once a threshold is reached, curcumin drives in parallel endoplasmic reticulum (ER) stress causing calcium release that can result in mitochondrial destabilization, producing more ROS and eventually overcoming the antioxidant defense system (ADS) of cells. This biphasic response is a key feature of an hormetic response discussed previously [2, 3, 20].

## 2. Cellular Uptake and Intracellular Distribution of Curcumin

**Figure 3:**
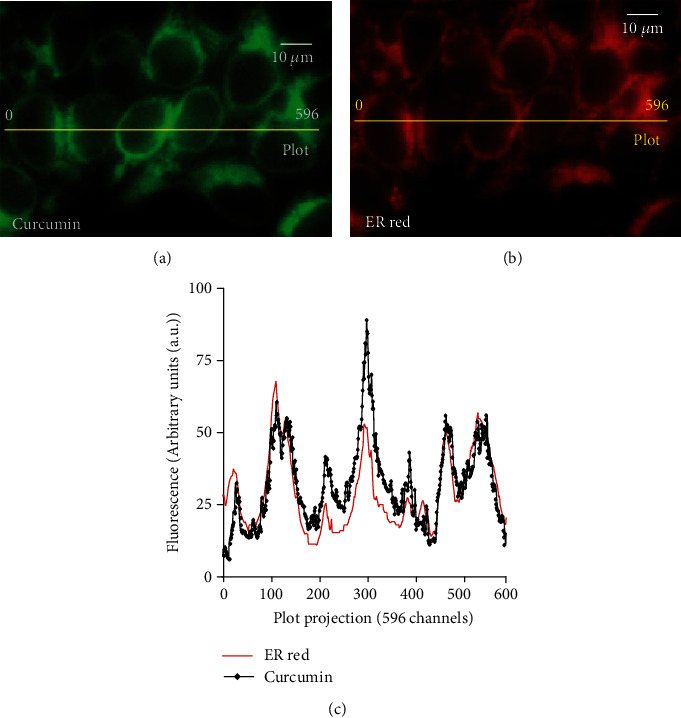
Curcumin localizes at the endoplasmic reticulum membrane (Patrice X. Petit, personal data and [2, 3]).

**Figure 4:**
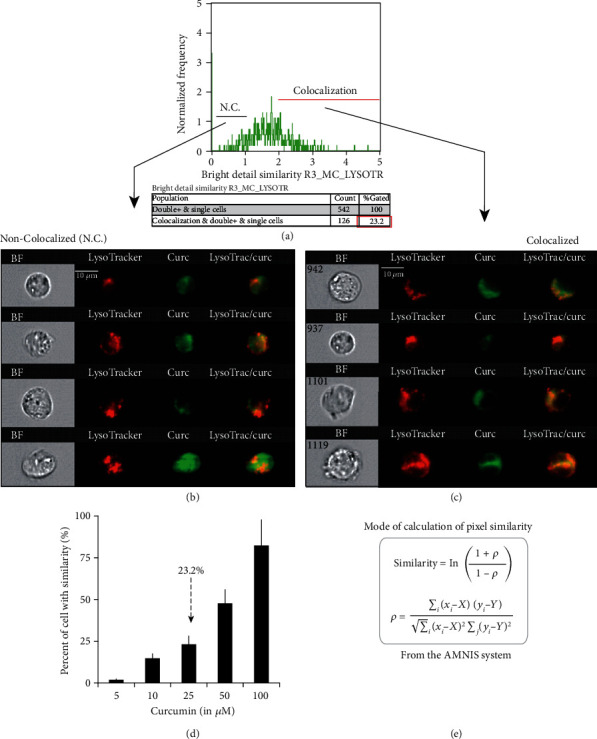
Lysosomal localization of curcumin as a function of the concentration. (a) Analysis of the colocalization of curcumin and LysoTracker Red (lysosomes) by using the Similarity Score included in IDEAS 6.0 software™ (Amnis®). This score, a log-transformed Pearson's correlation coefficient between the pixels of two image pairs, provides a measure of the degree of colocalization by measuring the pixel intensity correlation between the curcumin and LysoTracker images. Analysis was performed on 542 cells. Cells that were permeable to TO-PRO-3 iodide and/or debris were excluded from the analysis together with cellular aggregates. This corresponds to 5 *μ*M curcumin and 3 h incubation plus 10 min staining at 37°C with 100 nM LysoTracker Red. (b) Selection of some images that correspond to cells where there is no strict correlation between curcumin and LysoTracker Red. (c) Selection of some images corresponding to the cells presenting full colocalization of the two probes from the histogram in (a). (d) Percentage of cell population with a similarity index above 2. (e) Equation used for copixelisation analysis. Reproduced from Sala de Oyanguren [3].

Cellular distribution of curcumin has not attracted much attention, even though curcumin fluorescence can be used to localize it within cells, which is of great interest in understanding its mode of action. The effects of curcumin, including the drop in ΔΨ*m* and the production of ROS, do not appear to be the consequences of its direct action on mitochondria [2, 3].Indeed, the very early release of calcium into the cytoplasm following curcumin treatment led us to investigate potential interactions between curcumin and ER, which contains the main cellular pool of free calcium. Cellular uptake was observed by confocal microscopy in HuH-7 cells incubated with 20 *μ*M curcumin (without pH indicator) after different time intervals ranging from 0 to 48 hours.As shown in Figure 3, Curcumin fluorescence colocalized with ER red staining [3]. We also evaluated, by Amnis®, curcumin fluorescence with the pattern of lysosome staining assessed with LysoTracker Red DN99 and observed that some lysosomes presented both types of fluorescence, whereas others did not (Figure 4).

This suggests that curcumin interactions with lysosomes are strictly dependent on its concentration, a situation that can be explained by an additive pathway which may cooperate with the ER/calcium/mitochondrial pathway previously described [2, 3, 21].

## 3. Curcumin-Metal Complexation Reactions

The hydrogen bonding and hydrophobicity of curcumin associated with its aromatic ends and tautomeric structures along with the flexibility of the linker are responsible for noncovalent interactions. Curcumin forms strong complexes with most of the known metal ions [1]. The *α*,*β*-unsaturated *β*-diketo moiety of curcumin behaves as a chelating agent. Over the last decade, many papers have been published on metal-curcumin complexes, including examples of interactions [18, 19, 22–27] and review papers [1, 23]. Although it is well known that curcumin reduces metal toxicity in living systems through complexation, the actual role of these metal complexes of curcumin in cellular physiology is quite complex and unclear.

**Figure 5:**
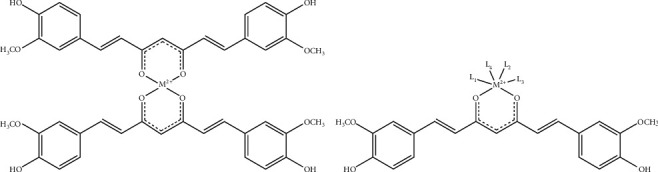
Reaction of curcumin with metals. (a) Structure of a 2 : 1 curcumin : metal complex. (b) Curcumin chelates metals with other ligands. Adapted with permission from Priyadarsini [1].

More precisely, curcumin can act as a chelating agent (presumably bidentate) for Fe^2+^ [28], Fe^3+^ [29, 30], and Cu^2+^ [14, 31] (Figure 5). As a chelator of iron, curcumin is supposed to alleviate H_2_O_2_ reduction, which produces hydroxyl radicals (HO^•^) and other ROS [28].

The *α*,*β*-unsaturated *β*-diketo moiety of curcumin forms chelates with transition metals, thereby reducing metal-induced toxicity, and some metal complexes exhibit improved antioxidant activity as enzyme mimics. Specific analogs are being developed to improve these activities and have been summarized recently [1, 32, 33].

In this broad literature, most of the promising medicinal applications of metal-curcumin complexes are in the field of anticancer activity with selective cytotoxicity and antineurodegenerative disorders with antioxidative/neuroprotective activity [34–38]. Curcumin-metal complexes not only modify the physicochemical properties of curcumin but also affect the biological reactivity of the metals. From our observations, the proautophagic and proapoptotic activities of curcumin [2, 39] are abolished by complexation [20]. Complexation with other metals like Cu^2+^ and Mn^2+^ can also reduce their toxicity, and some of the curcumin complexes behave as new antioxidants like superoxide dismutase mimics [14, 15, 17, 18, 31, 40, 41].

In fact, all the metals involved in Alzheimer's disease can form stable complexes with curcumin [18, 19, 42]. For example, curcumin forms three different types of complexes with Al^3+^, a major suspect in Alzheimer's pathophysiology. In 1 : 1 stoichiometry, the Al^3+^-curcumin complex has less DNA-binding affinity than free Al^3+^, which is recognized as a hallmark in reducing the development of Al^3+^-induced Alzheimer's disease [19, 42]. There are many other complexes: Ga^2+^-curcumin complexes developed as innovative bioceramics [43]; Zn^3+^-curcumin with anticancer, gastroprotective, and antidepressant effects in rats [44, 45]; Au^2+^-curcumin (five-coordinated form), which shows antiarthritic activity *in vivo* [46]; and vanadyl-curcumin ([VO(Cur)_2_]^2+^), which has antioxidant and antirheumatic activity [47]. Also, it is evident that curcumin reduces the toxicity of heavy metals like Hg^2+^, Cd^2+^, and Pb^2+^ through metal coordination, with significant reduction in oxidative stress [25, 48–51].

To our knowledge, curcumin-Fe complexes are unable to induce cell death as curcumin alone does [20]. What is truly interesting is that curcumin-metal complexes may also exhibit hormetic behavior, which extends the range of action of curcumin's biological activity [39].

## 4. Curcumin Reactivity with Reactive Oxygen Species

Curcumin, with its three functional groups—one diketone moiety and two phenolic groups, sustains many reactions like hydrogen donation leading to oxidized curcumin, reversible and irreversible nucleophilic addition, hydrolysis, or enzymatic reactions [32].

**Figure 6:**
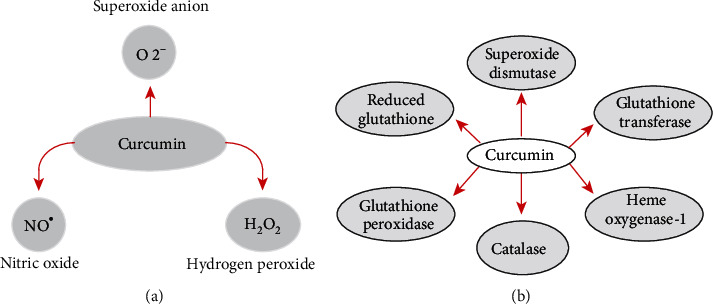
Curcumin antioxidant properties. (a) Curcumin can react directly with ROS. (b) Curcumin upregulates many components of the antioxidant defense system (ADS). Reproduced from Pavan et al. [210].

Curcumin is an excellent scavenger of most ROS, a feature that partially confers its antioxidant behavior in biological systems (Figure 6). ROS consist of both free radical oxidants and molecular oxidants [29, 52–58].

Free radical oxidants participate in electron transfer reactions and in hydrogen abstraction. All three active sites of curcumin may be oxidized by electron transfer and hydrogen abstraction. All three sites in curcumin can be oxidized, but the easiest abstractable hydrogen is from the phenol group, resulting in the formation of phenoxyl radicals, which are stabilized across the keto-enol structure. The most interesting example is the fact that peroxyl radicals (ROO^•^) can react with curcumin and form phenoxyl radicals that are less reactive than peroxyl radicals, thereby enhancing protection against ROS-induced oxidative stress. Soluble antioxidants like ascorbic acid confer upon the molecule a chain-breaking antioxidant capacity like that of vitamin E [53]. Curcumin scavenging of several other free radical ROS such as hydroxyl radicals, superoxide radicals, and alkoxy radicals has been described [53, 55, 57, 58]. The reaction of curcumin with superoxide radicals—generally produced at the inner mitochondrial membrane and not diffusible—is as efficient as other antioxidants and leads to catalytic degradation of superoxide in which curcumin acts as a superoxide dismutase mimetic [55].

ROS behavior and function are interestingly bizarre as they are the main regulators in the initiation and regulation of autophagy, cell survival, and cell apoptosis [59]. Despite that antioxidant activity in normal cells and prooxidant activity in cancerous cells have been established, there is still no clear biochemical explanation to this dual function of curcumin [1]. Nevertheless, this selective dual function suggests that curcumin is a potential adjuvant in chemotherapy and radiotherapy protocols to enhance cancer sensitivity and reduce toxicity to normal tissues [60–62].

In cancer cells, curcumin induces ER membrane destabilization, releasing Ca^2+^, activating downstream signaling proteins such as C/EBP homologous protein (CHOP), and upregulating ER transmembrane proteins (PERK, IRE-1*α*, ATF6) and proapoptotic Bcl-2 protein. These proteins are mediators of ER homeostasis, and excessive accumulation in the ER activates apoptosis [63, 64]. Over a certain threshold, curcumin induces ER-mediated apoptosis, while lower levels of ROS for the same pathway—under moderate stress like hypoxia—stay in the homeostatic range and allow cancer cells to duck apoptosis [65].

Because cancer cells maintain high levels of ROS—as a consequence of high ROS production or a decline of ROS scavenging capacity—they are selectively vulnerable to further ROS augmentation caused by an exogenous agent like curcumin [66]. Curcumin and new curcumin derivatives at high intracellular concentration (≥2.5 *μ*M) also behave like proapoptotic agents through mitochondria-dependent mechanisms that induce cell death (mainly apoptosis) in a wide range of cell types [67–71]. Furthermore, curcumin exerts antioxidant effects upon mitochondria via different mechanisms, i.e., decreased production of ROS and upregulation of antioxidant enzymes [72–75].

Enhanced ROS sensitivity of cancer cells could also be attributed to depletion of the reduced thioredoxin (Trx-SH) pool caused by the inhibition of thioredoxin reductase 1 (TrxR1) by curcumin [76]. Indeed, Trx-SH and TRxR1 are key mediators in the maintenance of redox homeostasis of cells by maintaining a defense pool against oxidation [77].

## 5. Curcumin at the Crossroads between Autophagy, Necroptosis, and Apoptosis

In the following sections, we will try to depict the mechanistic aspects of curcumin-induced autophagy and apoptosis separately, occurring in the general context of a complex intracellular machinery dysregulation. This approach is somewhat absurd, but will facilitate the depiction of parallel signaling pathways all of which have their own thresholds regarding mitochondrial life, autophagy, cell cycle arrest, and cell death.

### 5.1. Curcumin, Autophagy, and Cell Death

Autophagy is the degradation process of supernumerary or dysfunctional components within cells. Any targeted cytosolic materials or organelles are ultimately delivered and recycled in lysosomes thus functioning as an important biological mechanism for cell homeostasis. As autophagy is such an ubiquitous and fundamental mechanism for the cell, autophagy dysfunction can be found in a number of diseases even if molecular evidences are still needed [78]. Autophagy in cancer diseases has attracted much attention to modulate cell death when targeted by therapeutic candidates like curcumin.

With the discovery and characterization of Atg proteins, the suppressive function of autophagy in cancer has been validated [79, 80]. Among the Atg genes, beclin 1 (Atg6) is an essential tumor suppressor that modulates the initiation and regulation of autophagy. BECN1 gene deletion is often present in human breast, ovarian, and prostate cancers, and aging Becn1^+/-^ mice are prone to tumors including lymphomas and lung and liver cancers [81–83]. The accumulation of autophagosomes in dying cells is correlated with autophagic cell death, also defined as a nonapoptotic form of programmed cell death (PCD) or type II PCD with a potential function of tumor suppression similar to apoptosis.

In addition to its tumor suppressive role, autophagy is involved in cancer cell survival under stress. For example, immortalized, apoptosis-defective, IL-3-dependent bone marrow cells deprived of growth factor show a better survival response when autophagy is induced. Accordingly, autophagy inhibition accelerates cell death [84, 85]. Other stressors like hormonal deprivation, chemotherapy, and radiation—in many cases—upregulate autophagy as a cell survival mechanism [86]. As this upregulation can be cell specific and modulates cell fate in a tissue, it may contribute to treatment resistance. In tumors, inflammation and lack of vasculature may often result in a decrease of glucose and oxygen levels. This perturbation in the tumor microenvironment, as well as acidosis, can induce autophagy. Autophagy is therefore a functional driver and a marker of cancer severity in clinical research [86, 87].

In this context, the anticancer activity of curcumin can be investigated. Curcumin acts also both as a tumor suppressor and cancer cell protector [86–89].

The injection of curcumin in mice bearing breast cancer produces a clear inhibitory effect on the growth of breast cancer cells and metastasis [90].

**Figure 7:**
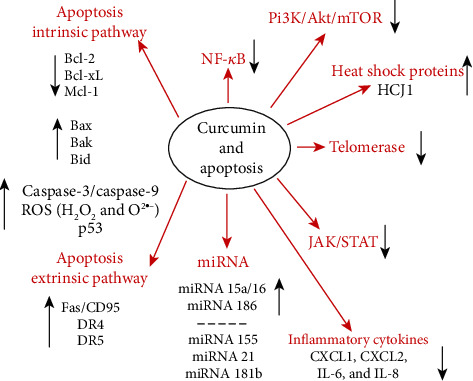
Schematic interpretation of the effects of curcumin on apoptosis. Representation is modified from Pavan et al. [138], redrawn, and completed. In red are the main pathways and proteins affected. In black, indirect interaction or tissue-dependent pathways.

Curcumin can inhibit the proliferation of tumor cells (Table 1) and induce the apoptosis of tumor cells (Figure 7), including bladder cancer [91], pancreatic cancer [92], prostate cancer [93], and uterine cervix carcinoma [94]. Curcumin also enhances the sensitivity to thermotherapy and *γ*-ray therapy [95, 96].

*In vitro*, curcumin inhibits cell proliferation of chronic granulocyte leukemia (CGL), glioblastoma, and esophageal cancer through autophagy induction, by upregulating LC3-II and beclin 1, as well as accumulating autophagosomes. In contrast, with the autophagy inhibitor bafilomycin A1, curcumin-induced cell death is inhibited [97].

Curcumin can inhibit both PP1 and the Akt/p70S6K pathway to activate extracellular signal-regulated kinases (ERK1/2) and finally induce autophagy [98]. Besides activating autophagy, curcumin also exhibits time- or concentration-dependent inhibition of the growth of K562 cells.

Cell death induced by curcumin is correlated with the generation of autophagosomes, a drop of mitochondrial potential, and caspase activation [2, 3, 39]. In addition, curcumin reduces the expression of Bcl-2 protein in K562 cells [99]. A combined treatment of curcumin and adriamycin enhances the apoptosis of HepG2 cells by reducing the proportion of Bcl-2/Bax protein and caspase-3 activation, in parallel with an increase in autophagic flux and mitochondrial fission. These data may indicate that curcumin can increase adriamycin-induced toxicity by activating mitochondria-mediated autophagy [100].

### 5.2. Curcumin, ER-Mitochondria, and Apoptosis

The initial effects of curcumin could be due to its interaction with subcellular compartments since, as a lipophilic polyphenol, it could be associated with total lipophilic load and membranes. These subcellular compartments are essentially ER and the lysosomes [2, 3, 39], since oxidative stress [101–104], lipid peroxidation [105], and calcium increase [106, 107] are associated with curcumin treatments and all these events are involved in the induction of cell death [102, 108] or specifically apoptosis [109–111]. A plethora of recent papers brought evidence of curcumin binding to and/or inhibiting numerous proteins, i.e., Nrf2, *β*-catenin, NF-*κ*B, inducible nitric oxide synthase, nitric oxide, amyloid plaques, ROS, cyclin D1, glutathione, cytosolic phospholipase A2, inhibitor of NF-*κ*B kinase-1-2, P_38_MAPK, p-Tau (p-*τ*), and TNF*α*.

Most of the mechanisms by which curcumin exerts its anticancer effect have been reported to be related to cell death induction. Curcumin inhibits the inhibitor of *κ*B kinase and I*κ*B*α* phosphorylation [112–115].

**Figure 8:**
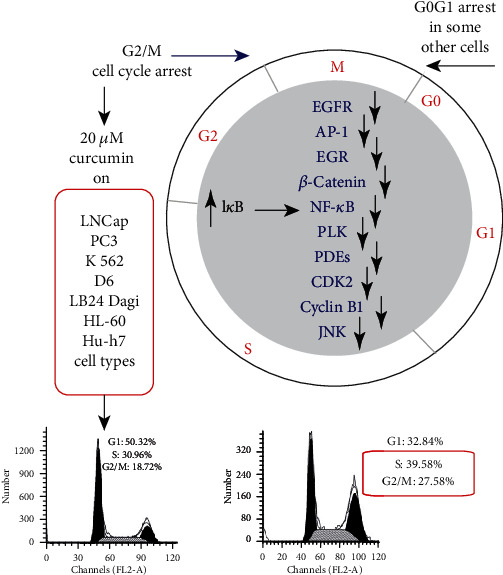
Schematic interpretation of curcumin interaction with partners involved in cell cycle and validation of these effects of G2/M blockade on many cell lines.

So, curcumin downregulates all genes downstream of NF-*κ*B such as Bcl-2, Bcl-XL, cyclin D1, cyclin B1, matrix metalloproteinase-9, cyclooxygenase-2, and interleukin-6, resulting in cell cycle arrest (G2/M) (Figure 8) and induction of apoptosis [64, 112, 115–117]. Also, curcumin exerts a strong inhibition resulting in a decreased cellular proliferation and induction of apoptosis that is mediated via the blockage of the Akt/mammalian target of rapamycin (mTOR) pathway and the phosphorylation of p70 ribosomal protein S6 kinase (p70S6K) and eukaryotic initiation factor 4E-binding protein [64, 118–120]. Among the antitumor effects of curcumin are the downregulation of the transcription factors activator protein-1 [121–125] and Egr-1 (Figure 8). Curcumin very potently reduces the cellular entry of viruses [126] and suppresses phorbol-ester-induced tumor promotion [127]. As curcumin can enter intracellular membranes and modify permeability and fluidity, it also acts on transporters and ion channels [128, 129]. Curcumin in association with the TORC1 and 2 inhibitors is thought to induce apoptosis via lysosome membrane permeabilization-associated autophagy [130].

-The curcumin capacity to induce an hormetic response is characterized by the numerous targets driving— depending on the dose —both antioxidant and prooxidant properties that can be related to the autophagic and cell death processes. The molecular circuits that link curcumin to cellular stress and death and how these pathways are uncoupled during hormetic responses are subjects of great interest [2, 3, 39]. Curcumin at very low concentrations (≤1 *μ*M) behaves as an excellent antioxidant, but higher concentrations of curcumin (5–10 *μ*M) operate primarily as an autophagy inducer, correlated with their capacity to reduce the acetylation of cytoplasmic proteins and cell cycle blockers (Figure 8).

Finally, at even higher concentrations—over 25 *μ*M—autophagy fails to rescue cells and cell death is induced. We investigated the mechanistic aspects of the destabilization of ER and lysosomes involved in mitochondrially associated apoptosis. Curcumin induces an ER stress causing calcium release that in turn destabilizes the mitochondrial compartment to induce apoptosis. These events are also associated with lysosomal membrane permeabilization and activation of caspase-8, mediated by the activation of cathepsins and calpains [2, 3]. This complex interplay is of huge interest, as efficient autophagy may allow cells to escape the G2/M blockade [2] induced by curcumin when used at around 10-20 *μ*M in the extracellular medium [3] (Figure 8).

### 5.3. Curcumin, Lysosomes, and Autophagy

As mentioned before, under physiological conditions, basal autophagy is a catabolic process where lysosomes are mainly involved in the degradation of damaged components and dysfunctional organelles in cells. Autophagy has attracted the interest of scientists in the field of cancer research because it is designated as an alternative programmed cell death (type II), whereas apoptosis is well known as programmed cell death type I [131]. But this definition has been modified and adapted to the discovery of multiple cell death pathways and of a diversity of autophagic processes.

We previously showed that a fraction of internalized curcumin is bound to the lysosomal membranes [2, 3] (Figure 4). Lysosomal destabilization by curcumin is critically dependent on the intracellular curcumin concentration; the occurrence of soluble lysosomal hydrolases, i.e., cathepsins and chemotrypsins; and the dysfunction of lysosomal-associated membrane proteins (LAMPs).

Growing evidence argues for the presence of highly activated PI3K/Akt signaling in cancer cells compared to normal ones. As we said earlier, curcumin inhibits the Akt-mTOR pathway and interferes with PI3K/Akt signaling, leading to the inhibition of the proliferation and reduction of the invasiveness and migration of various cancer cells, including triple-negative cancer cells [132–135].

The Akt/mTOR/p70 ribosomal protein S6 kinase (p70S6K) and the extracellular signal-regulated kinase 1/2 (ERK1/2) pathways are two major pathways that regulate autophagy induced during nutrient starvation. The Akt/mTOR/p70 and ERK1/2 pathways are frequently associated with oncogenesis in a variety of cancer cell types, including malignant gliomas. In U87-MG and U373-MG malignant glioma cells, curcumin induces G2/M arrest (Figure 8) and nonapoptotic autophagic cell death. It inhibits the Akt/mTOR/p70S6K pathway and activates the ERK1/2 pathway, thus inducing autophagy. It is interesting that the activation of the Akt pathway inhibits curcumin-induced autophagy and overall cytotoxicity, whereas the inhibition of the ERK1/2 pathway inhibits curcumin-induced autophagy and induces apoptosis, thus resulting in enhanced cytotoxicity. These results suggest that curcumin has high anticancer efficacy *in vitro* and *in vivo* by inducing autophagy [98].

Nevertheless, the effect of curcumin on lysosomes remains largely elusive. Some recent data suggest that currently known transcription factor EB (TFEB) activators are mainly inhibitors of mTOR which, as a master regulator of cell growth and metabolism, is involved in a wide range of biological functions [132–137] and exerts its effect at the lysosomal membrane surface [137].

It has also been suggested that curcumin treatment may activate TFEB [138]. TFEB is a major player of the transcriptional response to starvation and controls autophagy by inducing lysosomal biogenesis, regulating autophagosome formation and autophagosome-lysosome fusion both *in vitro* and *in vivo* [139]. This is based on the cardinal hypothesis that a lysosome-to-nucleus signaling mechanism senses and regulates the lysosome via TFEB and mTOR [140]. Linked to lysosomal membranes, TFEB colocalizes with the master growth regulator mTOR complex 1 (mTORC1). The arguments for the fundamental role of TFEB are the following: curcumin binds directly to TFEB (and/or disturbs the membrane in the vicinity of TFEB insertion), promotes TFEB nuclear translocation, and increases the transcriptional activity of TFEB. TFEB modulators that act without inhibiting the mTOR pathway would probably be less deleterious to cells [141]. Along with this new argument, it has been reported that curcumin does not inhibit mTOR and fails to activate lysosomal function when constitutive activation of mTOR has been engineered, proving that curcumin-mediated lysosomal activation is achieved via suppression of mTOR activity. The AMPK-JNK pathway can also be activated by curcumin, which drives both mTOR inhibition and Bcl-2 upregulation and in turn enhanced autophagy and suppressed apoptosis [142]. Finally, inhibition of autophagic fluxes and activation/destabilization of the lysosomal compartment by curcumin, if passing a certain threshold, lead to more cell death, suggesting that lysosomal activation and enhanced autophagy serve—if successfully executed—as a cell survival mechanism to protect against curcumin-mediated cell death.

Taken together, all these data built up a novel insight into the regulatory mechanisms of curcumin at the lysosomal level (enhancing autophagy), which may reinforce the relevance of curcumin as a potential cancer therapeutic agent [138], but may also be used for lysosomal storage disorders, neurodegenerative disorders, and cardiovascular diseases.

## 6. Curcumin and Mitochondrial Turnover

### 6.1. Mitochondrial Biogenesis

Mitochondria are the key compartments of cellular energy metabolism that is also fundamental for apoptosis regulation and cell signaling. It is now established that the mitochondria of malignant cells differ structurally and functionally from those in normal cells and are mainly characterized by ROS overproduction, which may promote genomic instability by the alteration of gene expression and the modulation of signaling pathways. Both oxidative damages targeting the mitochondrial compartment and nuclear DNA induce further alterations of oxidative phosphorylation and enhance mitochondrial-specific ROS production, sustaining a “vicious cycle” between mitochondrial ROS, genomic instability, and cancer development. Alternatively, an impaired oxidative phosphorylation or mitochondrial biogenesis is found in neurodegenerative diseases [143–145], cardiovascular diseases [146], and type II diabetes [147].

In this context, mitochondrial biogenesis—taken as the increase of the mitochondrial network—is a complex response to various stimuli that relies on both mitochondrial and nuclear genomes. Usually stimulated under supraenergetic demand, mitochondrial biogenesis involves a master regulator called peroxisome proliferator-activated receptor *γ* coactivator 1-*α* (PGC-1*α*) with peroxisome proliferator-activated receptor alpha (PPAR*α*) activating nuclear respiratory factors 1 and 2 (NRF1/NRF2) among others [148]. Metabolic sensors like AMP-activated protein kinase (AMPK), NAD^+^/NADH′H^+^ ratio, or ROS levels are first players of the PGC-1*α* pathway [149]. AMPK can modulate NAD^+^ levels in the cell contributing to [150] the activation of a NAD^+^-dependent deacetylase sirtuin 1 (SIRT1), which activates PGC-1*α* through deacetylation [151–153]. Accordingly, high PGC-1*α* levels are found in tissues with high rates of oxidative phosphorylation, facing high ATP needs [154–156]. Once activated, the AMPK/SIRT1/PGC-1*α* pathway involves NRF1 and NRF2 activating downstream of the estrogen-related receptor *α* (ERR*α*) and the expression of mitochondrial transcription factor A (TFAM) and transcription factors B1 and B2 (TFB1M and TFB2M) [157–163]. These transcription factors are responsible for both the increased expression of nuclear DNA encoding for mitochondrial proteins [164] and the transcription/replication of mitochondrial DNA (mtDNA) [165–167], thereby orchestrating mtDNA homeostasis at large [165, 168, 169].

**Figure 9:**
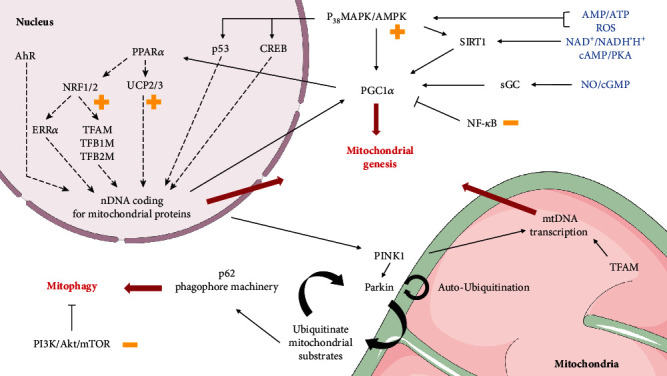
Mitochondrial turnover highlights with curcumin regulations. Various energetic and redox sensors (in blue) can activate the master regulator PGC-1*α* stimulating mitochondrial biogenesis mainly by upregulating transcription of numerous mitochondrial proteins. Plus and minus yellow signs indicate curcumin regulations. Nonselective mitophagy can be activated by various pathways like mTORC1, but selective mitophagy is initiated by depolarized mitochondria. Loss of mitochondrial membrane potential is followed by PINK1 accumulation at the surface which phosphorylates PARKIN. Active PARKIN further promotes its recruitment and phosphorylation by PINK1 in a feed-forward mechanism. These ubiquitinated substrates can activate p62 and the autophagy machinery to perform mitophagy. Some graphic elements are adapted from Servier Medical Art (CC-BY 3.0).

In this context, the AMPK/SIRT1/PGC-1*α* pathway orchestrates mitochondrial biogenesis and participates in redox homeostasis, crucial for cell life (Figure 9).

Studies describing curcumin as an enhancer of mitochondrial biogenesis are quite recent and should point to a new path for curcumin studies, as a kick-start to develop newly designed drugs that target dysfunctional mitochondria found in cardiovascular diseases and neurodegeneration.

However, the external concentrations of curcumin used in experimental models discussed here are high and may not be reached in the human system through food or oral medication. As mentioned before [2, 3], it will be necessary to improve curcumin bioavailability, to ensure that higher curcumin concentrations can be reached, or to find a way to address curcumin to target cells. This explains why curcumin is becoming a common model in biotechnology and drug delivery studies seeking better permeability and stability [170] as well as new nanoformulations [170–172].

*In vitro*, curcumin has been described as a potent inducer of a brown fat-like phenotype in 3T3-L1 and primary white adipocytes [173].

In these cells, 20 *μ*M curcumin for 6 days upregulates PGC-1*α*, associated with an increased level of cytochrome *c*, the phosphorylated form of AMPK (p-AMPK), Nrf1, TFAM1, and the total mtDNA transcript.

In gentamicin-treated LLC-PK1 cells, 30 *μ*M of curcumin for 24 h induces Nrf2 translocation and upregulates PGC-1*α*. This evidence shows that curcumin can elicit mitochondrial biogenesis *in vitro* via Nrf2 as a master regulator of the redox cellular environment [174, 175].

More *in vivo* studies are in line with these data and show that curcumin can upregulate respiratory chain components. In gastrocnemius and soleus muscles of rats submitted to long-lasting exercise training, curcumin induces an increase in the amounts of cytochrome *c* oxidase and various subunits of complexes I, II, and III [176]. Curcumin also stimulates the effects of exercise training upon mtDNA synthesis and citrate synthase (CS) activity which are the gold standards for evaluating mitochondrial biogenesis in muscles. As curcumin can modulate AMP and NAD^+^ levels, it can thereby activate AMPK and all downstream players of the AMPK/SIRT1/PGC-1*α*. Rats submitted to ischemic reperfusion injury after curcumin pretreatment exhibited a better developed mitochondrial network in the cerebral cortex [177]. Curcumin pretreatment prevented the injury-induced downregulation of uncoupling protein 2 (UCP2),Nrf1 levels. and TFAM which were upregulated at all concentrations tested. Curcumin (400 mg/kg day^−1^) provided by gavage prior to gentamicin (a renal toxin) exposure preserved mitochondrial morphology and enhanced the number of mitochondria [175].

In mice, curcumin also demonstrated the browning of white adipose tissue by UCP1 and PGC-1*α* upregulation, with an increase of mtDNA transcripts [178].

All these findings reinforce the new hypothesis that curcumin at very low concentrations may induce mitochondrial biogenesis, but new data on the impact of curcumin on regulators of mitochondrial biogenesis, such as AMPK, SIRT1, NRF1, and TFAM, will be of great interest.

### 6.2. Curcumin and Mitophagy

It is now established that a fine regulation balancing mitochondrial biogenesis and mitophagy is essential to maintain the adaptability of the cell to its metabolic state, intracellular stress, and environmental signals [179].

Regulatory factors contributing to mitochondrial homeostasis have also been linked to carcinogenesis, which points at mitophagy as a potential target for cancer therapy [180].

Mitophagy is the process by which damaged mitochondria are removed from the cells through engulfment by an active autophagosome in a PTEN-induced kinase 1 (PINK1)/Parkin (E3 ubiquitin ligase)-dependent mechanism [181–183]. Despite being a physiological process, increased rates of mitophagy have been found in some human diseases and may represent a major risk for redox and bioenergetic homeostasis [184–187].

The capacity of curcumin to induce mitochondrial biogenesis (certainly at low concentrations) in response to enhanced energetic demand is followed by curcumin's potential to trigger mitophagy [2, 3, 188], and once again the threshold concept is of most relevance.

CNE2 cells (nasopharyngeal carcinoma) can be sensitized with 10 *μ*M curcumin before being exposed to ultrasound [189]. A combination of ultrasound and curcumin increases the number of swollen mitochondria and impairs mitochondrial membrane architecture. These results constitute some evidence that altered mitochondria could be eliminated by specific autophagy in response to curcumin. Nevertheless, more data are needed to elucidate if curcumin is a general inducer of autophagy or contributes specifically to a mitophagic process.

Additional data are needed to elucidate the exact role of curcumin as an inducer of mitophagy. The scenario in which curcumin would be useful in inducing mitochondrial degradation requires more detail.

## 7. Curcumin and Regulatory MicroRNA

In cancer research, microRNAs (miRs) have attracted much attention as one therapeutic agent could physiologically regulate multiple targets to limit cancer progression. However, as multiple pathways are involved with hundreds of miRs, this approach is still challenging. Recent studies revealed that curcumin can regulate miRs, and one strategy may be to investigate miRs through curcumin multitargeting.

MicroRNA-21 (miR-21) can be taken as a model for studying regulatory mechanisms between microRNA and curcumin [190, 191]. miR-21 is involved in proliferation, apoptosis, metastasis, and anticancer drug resistance. miR-21 is also involved in several downstream pathways, such as phosphatase and tensin homolog (PTEN), phosphoinositide 3-kinase, protein kinase B (PI3K/Akt), programmed cell death protein 4 (PCD4), and MAPK pathways, and in the enhancement of p53 and NF-*κ*B pathways. It is interesting to note that all these pathways have been described as being affected by curcumin cellular loading. Curcumin decreases miR-21 levels by both increasing the miR-21 exosome outside the cells and by binding to its promoter, a situation that decreases the transcription of the miR-21 gene.

Beyond miR-21 inhibition, curcumin induces epigenetic alterations by modulating the expression of several other oncogenic and tumor suppressor miRs (Table 2). Suppression of oncomiRs such as miR-21, miR-17-5p, miR-20a, miR-27a, and miR-186∗ [191] and overexpression of miR-34 and epithelial-mesenchymal-transition suppressor miRs are among the most important effects of curcumin [192]. A recent publication highlights curcumin as a relevant miR regulator for cancer progression and points out that exosomes produced in curcumin-treated cells contain both miRs and curcumin and carry anticancer properties for the recipient cells [193]. This mechanism of physiological vectorization of curcumin, by curcumin or not, may open a new approach to investigate for potential therapeutic tools in cancer therapy.

## 8. Curcumin: PAINS or Nutraceutical?

As previously discussed, curcumin has been attracting massive attention, particularly over the last decade, not only for its use as a chemotherapeutic agent but also for its antioxidant properties. It has several advertising advantages: a natural product already used in Ayurvedic medicine, easily extractible from plants (turmeric), rather inexpensive, and with beneficial effects when included in the diet, the latter observation being initially supported by epidemiological studies. Its pleiotropic effects as evidenced in numerous studies attracted the attention of scientists and lay people drawn to natural products now labeled as “superfoods.”

On the other hand, some researchers raised concerns about curcumin and other natural products classifying them as Pan Assay Interference Compounds (PAINS) [194].

Typically, it does not behave in a drug-like manner with its target, a behavior potentially unspecific, unquantifiable, and interfering with assays measuring them or other readouts. Warnings come from the drug discovery field, in high-throughput screening (HTS) where these PAINS largely return as “hits” for a considered target. Of course, anyone actively involved in high-throughput screening (HTS) should consider the latest considerations regarding PAINS to filter their hits [195]. In fact, HTS experts working with biologists know that it is considered a rather uncertain research often leading to multiple “hits”, hitting themselves multiple targets [196].

Medicinal chemists warned us about curcumin's status not only as a PAINS but also as an AIC (assay interference compound) and an IMP (invalid/improbable metabolic panacea) status) [197] which has led to divisions within the scientific community [198, 199]. The complexity of curcumin's behavior should reinforce our interest to discuss the best way to design our tests and readouts. In this review, we point out the hormetic behavior of curcumin and its ability to hit multiple pathways, and not only proteins but also membranes. What could be a “no go” to researchers following the perspective of one drug and one protein with one active site can be put in perspective of the advantages of modulating a balance of pathways and doing some, what researchers call, multitargeting [200].

To circumvent some of the limitations of natural products like poor solubility and bioavailability, researchers vectorize them with nanomaterials. A literature search will show that curcumin and nanoparticles return hundreds of studies, and more than half of them have applications for cancer; the rest are comprised of applications for inflammation and vascular diseases.

It would need a complete separate review to cover curcumin and its nanoformulations, but the therapeutic use of natural products could benefit from research in nanomedicine as some of the latest nanoformulations are using targeting moieties that are tissue- and cell-specific [201, 202]. Such nanoformulations could alleviate most of the systemic limitations of curcumin and bring that “multitargeting drug” at the cellular level.

## 9. Conclusion

Although some fourteen thousand publications contain the term curcumin, few studies have followed new research directions. It is clear that low levels of curcumin enhance mitochondrial biogenesis in cells and tissues, mainly through the induction of the PGC-1*α*-related signaling pathway [203, 204]. The exact mechanism of curcumin-induced mitochondrial biogenesis is incompletely understood, since the roles of AMPK, NRF1, Nfr2, and/or TFAM that are essential in biological processes were not investigated in most published studies. It is sure that TFEB is also involved in such pathway, participating in lysosomal biogenesis together with autophagy induction [138]. Molecules such as PGC-1*α* are thought to be critical for the maintenance of organelle content. The hypothesis that the induction of mitochondrial biogenesis by exogenous polyphenols may play a role in alleviating cellular dysfunction with the disruption of mitochondrial bioenergetics, in cases of neurodegenerative and cardiovascular diseases, is of great value, even though our knowledge of curcumin-mediated regulation of mitochondrial biogenesis is limited [165, 166, 168].

Investigations of the role of curcumin as an inducer of mitochondrial biogenesis and mitophagy in a context of mitochondrial energetic disturbance are in their infancy. By focusing efforts on TFEB, a protein widely considered to be the most important regulator of autophagy and lysosomal biogenesis, we may perhaps shed light on the regulation of mitophagy in various cell types.

Curcumin induces crosstalk between apoptosis and autophagy and is thought to interact with proteins that belong to both pathways and are involved in the regulation of cancer cell death. Most researchers consider that cancer progression is in large part due to defects in cell death mechanisms [205, 206]. These defects shield tumor cells from drugs and therapies, thus prolonging cell life and promoting cell dispersion. Autophagy and apoptosis are safeguards against cellular damages and uncontrolled outgrowth and differentiation of harmful cells. Autophagic proteins generally hinder apoptosis, whereas apoptotic intermediates prevent autophagic responses. Additionally, curcumin analogs have been described with attenuating properties on AD accumulation in a mouse model of Alzheimer's disease [207]. This effect is mediated by a reduced level of amyloid-*β* protein associated with enhanced autophagy.

**Figure 10:**
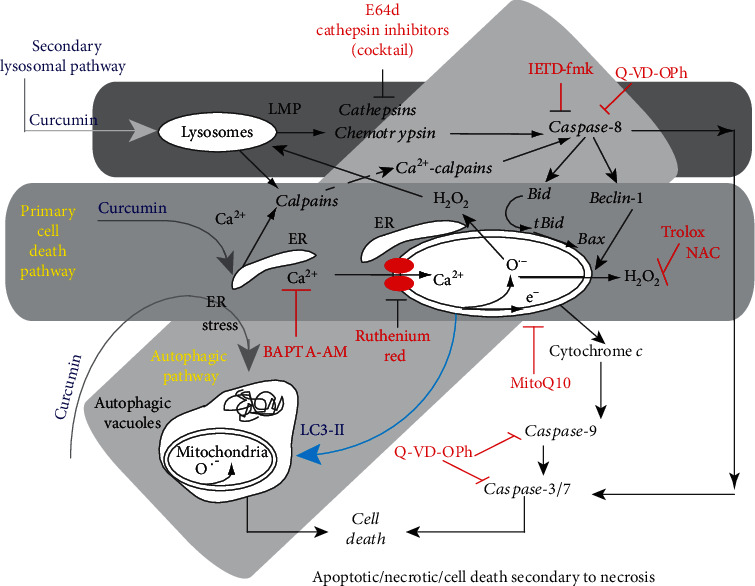
Crosstalk between apoptosis and autophagy in cells treated with curcumin. Curcumin mainly targets the endoplasmic reticulum (ER) and lysosomes. The classic apoptotic pathway is mediated by calcium release from the ER. Uptake of this calcium by mitochondria disrupts mitochondrial homeostasis. Calcium alters mitochondrial electron transport causing substantial ROS production (both superoxide anions and hydrogen peroxide), which leads to the opening of the permeability transition pore in the mitochondrial membrane. Consequently, cytochrome *c* is released and the caspase-9 and caspase-3/7 pathway is activated leading to cell death. Furthermore, the ER stress pathway leads to the formation of autophagic vacuoles that attempt to eliminate the dysfunctional mitochondria. The cleavage of beclin-1 is associated with early apoptosis and leads to the accumulation of autophagic vacuoles. So, despite the activation of autophagy, cells undergo a type of “necrotic cell death” following these initial apoptotic events. These two pathways are in parallel to a lysosomal pathway that is dependent on curcumin concentration (see Figure 4). Curcumin destabilizes lysosomal membranes leading to lysosomal membrane permeability and the activation of both cathepsins and chemotrypsins. Activated caspase-8 leads to beclin-1 cleavage that inhibits the primarily induced autophagy. The increase in cytosolic calcium concentration also activates calpains, which contribute to the degradation process and accelerate cell death. The various inhibitors used in this work are indicated in red at the place where the pathways are affected ([2, 39]; and Patrice X. Petit personnal communication). Big gray arrows indicate the entrance of the three main pathways that interfere (primary cell death pathway, secondary lysosomal cell death pathway, and autophagic pathway).

The case of curcumin, autophagy, and apoptosis, which are highly intricate pathways (Figure 10), can be investigated by manipulating their mutual proteins. Targeting these shared proteins involved in the crosstalk between autophagy and apoptosis so as to regulate tumor cell death is crucial for the successful design of future anticancer therapies. MicroRNAs are involved in the modulation of these components that interact in both autophagy and apoptosis in cancer cells. Since the development of miRNA-based therapeutics seems to be hazardous and time-consuming, additional approaches need to be considered. Curcumin as a phytochemical may fill this gap since it has been reported to switch these interplaying proteins to maximize cancer cell death through the partnership of autophagy and apoptosis [2, 39, 138]. This may enable us to chart the missing links between these machinery proteins, organelle membranes, and miRNAs.

Beyond curcumin, knowledge of the role of natural chemopreventive agents in autophagy and apoptosis will certainly expand [208] and may lead to anticancer therapies with minimal adverse effects [209]. The study of the role of natural agents that induce beneficial cross-talk between apoptosis and autophagy to finely tune cell death is also at the forefront of new therapeutic discoveries in metabolic disorders and aging.

## Figures and Tables

**Table 1:** Antiproliferative target for curcumin.

Target	Effect	Cancer type	Refs.
GRP78	Downregulation	Colon	[211]
EphA2	Downregulation	Melanoma	[212]
SOCS1 and 3	Upregulation	Leukemia	[213]
Nfr2	Downregulation	Breast	[214]
MiR15a/16-1	Downregulation	Leukemia	[215]
DCLE1	Upregulation	Colon	[216]
Skp2	Downregulation	Glioma	[217]
FOXO1	Upregulation	Pancreas	[218]
EZH2	Downregulation	Breast	[219]

**Table 2:** Curcumin alters miRNAs and relevant target expression in pancreatic, colorectal, breast, and lung cancers.

Cancer origin	Upregulated	Downregulated	Targets	Refs.
Pancreas	miR-22	miR-21	SP1, ESR1	[220]
	miR-200	miR-199a^∗^	PTEN	[221]
Colorectum	—	miR-21	AP1, Pdcd4	[222]
Breast	miR-15a	—	Bcl-2	[223]
	miR-16	—		
Lung	mir-206	—	PI3K/AKT/mTOR	[224]
Lung	miR-186^∗^	—	Caspase-10	[191]
Retinoblastome	—	miR-99a	JAK/STAT	[225]
Thymic carcinoma		miR-27a	Notch1/mTOR	[226]
Osteosarcoma	—	miR-21	RECK	[227]
Retinoblastome	miR-22	—	Erbb3	[228]

^∗^Additionally, curcumin regulates many other miRNA expressions, e.g., miR-1, miR-7, miR-9, miR-19, miR-34a, and miR-181 [193].

## Abbreviations

AP1: Activator protein 1 (AP-1), a transcription factor
Caspase-10: Cleaves and activates caspase-3 and caspase-7, and the protein itself is processed by caspase-8
EphA2: EPH receptor A2 (ephrin type-A receptor 2) is a protein encoded by the *EPHA2* gene and belongs to the ephrin receptor subfamily of the protein-tyrosine kinase family
EZH2: Enhancer of zeste homolog 2 is a histone-lysine N-methyltransferase enzyme (EC 2.1.1.43) encoded by the EZH2 gene which participates in DNA methylation and transcriptional repression
ESR1: Estrogen receptor 1
FOXO1: Forkhead box protein O1, also known as forkhead in rhabdomyosarcoma, is a protein that in humans is encoded by the *FOXO1* gene
GRP78: Member of the HSP family of molecular chaperones required for endoplasmic reticulum integrity and stress-induced autophagy
Nfr2: Nuclear factor (erythroid-derived 2)-like 2, also known as NFE2L2, is a basic leucine zipper (bZIP) protein that regulates the expression of antioxidant proteins that protect against oxidative damage triggered by injury and inflammation
Pcdc4: Programmed cell death protein 4 target of the oncomiR miR-21
PTEN: Gene that was identified as a tumor suppressor that is mutated in a large number of cancers at high frequency
Skp2: S-phase kinase-associated protein 2
SOCS1 and SOCS3: Genes that encode members of the STAT-induced STAT inhibitor family (SSI), also known as suppressors of cytokine signaling (SOCS)
SP1: Protein encoded by this gene is a zinc finger transcription factor that binds to GC-rich motifs of many promoters.
